# Novel edible coatings pretreatment for enhancing drying performance and physicochemical properties of cherry fruits during multi-frequency ultrasonic vacuum far infrared radiation — Radio frequency vacuum segmented combination drying

**DOI:** 10.1016/j.ultsonch.2025.107306

**Published:** 2025-03-12

**Authors:** Zepeng Zang, Xiaopeng Huang, Guojun Ma, Fangxin Wan, Yanrui Xu, Qiaozhu Zhao, Bowen Wu, Hongyang Lu, Zelin Liu

**Affiliations:** College of Mechanical and Electrical Engineering, Gansu Agricultural University, Lanzhou 730070, PR China

**Keywords:** Cherry, Multi-frequency ultrasonic, Edible coatings pretreatment, Radio frequency vacuum drying, Segmented combination drying

## Abstract

To maximize the drying efficiency and physicochemical quality of cherry fruits while minimizing energy consumption, this study investigated the effects of novel edible coatings (sodium carboxymethyl cellulose (CMC-Na) and sodium alginate (SA)) pretreatment combined with multi-frequency ultrasonic vacuum far infrared radiation-radio frequency vacuum (MUSVFIR-RFV) segmented drying on the drying performance and physicochemical properties of cherries. Results demonstrated that MUSVFIR-RFV segmented drying combined with coating pretreatment reduced the drying time by 11.11 ∼ 25.93 % compared to single drying. At a moisture conversion point of 50 %, the process achieved optimal drying performance and energy efficiency. Remarkably, multi-frequency ultrasound outperformed single-frequency ultrasound in terms of energy transfer intensity and uniformity. Physicochemical quality analysis revealed that the combination of CMC-Na or SA coatings with MUSVFIR-RFV segmented drying significantly improved the retention of soluble solids, individual sugar, natural bioactive compounds, TPC, TFC, and antioxidant activities (DPPH, ABTS, and FRAP). Texture and sensory properties showed that the hardness and adhesiveness of coated cherries were reduced, while elasticity, chewiness, and cohesiveness were significantly enhanced. Cherries subjected to (CMC-Na)-(MUSVFIR-RFV) treatment achieved higher scores in texture, crispness, color, sweet taste, appearance, and aroma, with lower bitterness and off-odor, leading to an overall acceptance score of 9.2, which was significantly higher than that of the control. Hierarchical clustering and PCA analysis further validated that the integration of coatings with segmented drying effectively improved the physicochemical quality of dried cherries. The findings provide scientific evidence for the development of efficient drying technologies for cherry, substantiating the potential advantages of combining edible coatings with MUSVFIR-RFV drying in enhancing drying efficiency, quality, and sensory attributes of cherries.

## 1 Introduction

Cherries (*Prunus pseudocerasus* (Lindl.) G. Don), primarily cultivated in North America, Europe, Asia, and other temperate regions, are nutrient-dense berries rich in sugars, amino acids, anthocyanins, vitamin C, and various essential trace elements, known as the “King of natural VC” and the “Fruit of life” [1,2]. However, due to their high water content and vigorous respiratory metabolism, the water activity in fresh cherries significantly accelerates enzymatic reactions and microbial growth. This leads to rapid quality deterioration, including moisture loss, wrinkling, softening, browning, fermentation, and mold growth during sales, transportation, processing, and storage, ultimately causing substantial economic losses [3,4]. In recent years, based on the demand for high-quality products and the fast-paced modern lifestyle, ready-to-eat fruit snacks, such as crispy fruit chips and preserved fruits, have gained popularity among consumers for their unique crispy texture and rich nutritional value. Drying, as a critical step in the high-value utilization of agricultural products, plays a vital role in ensuring food safety, enhancing flavor and texture, and extending shelf life [5,6]. By removing water from products and inhibiting microbial growth and enzymatic activity, drying extends the shelf life of agricultural products, increases their added value, and reduces transportation costs [7].

However, drying is an inherently complex, energy-intensive process characterized by highly nonlinear couplings of heat and mass transfer [8]. For cherries, which have high water content and are sensitive to heat, conventional single drying techniques face several challenges, such as low drying efficiency, severe nutrient loss, high energy consumption, and suboptimal product appearance and texture [9,10]. To meet modern consumer demands for efficient, energy-saving, green, safe, and low-carbon drying processes, the development of advanced combined drying technologies to improve the quality, flavor, and economic value of dried cherries has become a key focus in current research.

Segmented combination drying is an innovative drying technology that integrates two or more drying methods in a sequential manner [11]. This approach effectively combines the advantages of various drying techniques, enhancing drying efficiency, optimizing energy utilization, improving product quality, and minimizing stress generated during the drying process. As a novel non-ionizing electromagnetic radiation technology, radio frequency vacuum drying (RFV) synergistically integrates the benefits of radio frequency (RF) heating and vacuum drying [12]. Unlike traditional heating mechanisms that rely on internal conduction and surface convection, RF technology penetrates directly into the material's interior, inducing high-speed motion of polar molecules and charged ions [13]. This motion generates friction among molecules, rapidly converting electrical energy into thermal energy, thereby enabling rapid internal heating and efficient dehydration of the material. Additionally, in a vacuum environment, the boiling point of water is reduced, not only mitigates thermal damage to bioactive compounds but also markedly improves drying efficiency [14,15]. Hence, RFV technology has been increasingly applied to the postharvest dehydration of agricultural products. Nevertheless, despite its extensive application in agricultural product processing, RFV drying still faces certain technical limitations and challenges. Studies have shown that single-stage RFV heating, due to rapid temperature rise, may lead to edge effects and uneven heating, reducing the efficiency of thermal processing in agricultural. Moreover, such conditions may result in product scorching and flavor degradation. Similar findings have been reported by Mao et al. [16] and Ai et al., [17] who concluded that compared to single-stage RF drying, multi-stage drying approaches can overcome these drawbacks, enhance drying uniformity, and improve product quality.

Multi-frequency ultrasonic vacuum far infrared–radio frequency vacuum (MUSVFIR-RFV) segmented combination drying is an advanced hybrid drying technology. This method achieves efficient drying by leveraging MUSVFIR drying in the initial stage to rapidly remove a significant portion of free water while enhancing heat and mass transfer efficiency, followed by RFV drying to remove residual free and bound water effectively. Among these, ultrasound, as a high-frequency mechanical wave exceeding 20 kHz, is an emerging environmentally friendly non-thermal processing technology [18,19]. During the drying process, ultrasound interacts with the material medium to produce thermal, mechanical, and cavitation effects, thereby enhancing heat and mass transfer properties [20]. The thermal effect involves the conversion of ultrasonic energy absorbed by the material into heat, elevating the material's temperature. The mechanical effect, through high-frequency vibrations, causing the material to undergo repeated compression and tension. When the structural effect force exceeds the surface adhesion of water on the material's surface, water is effectively removed [21]. The cavitation effect arises from the periodic expansion and compression of cavitation bubbles induced by ultrasound, which ultimately collapse, generating localized high temperature, high pressure, micro-jets, and shock waves that further facilitate moisture migration and removal [22,23]. Additionally, far infrared radiation drying is widely used in food processing due to its high heating efficiency, uniformity, and low energy consumption. Unlike conventional convective drying, far infrared radiation transfers heat internally to the material, aligning the moisture evaporation direction with the heat transfer direction to achieve uniform heating [24]. Consequently, MUSVFIR-RFV represents a high-efficiency, energy-saving, environmentally friendly hybrid drying technology that effectively preserves product quality. It offers substantial potential for applications in food processing and dehydration of agricultural product.

Recently, due to consumer concerns about chemicals usage and legal restrictions, research on edible coatings has increasingly focused on the applying natural compounds to effectively preserve the physicochemical quality of fruits during storage [25]. Edible coatings are non-toxic, renewable, and biodegradable food-grade films applied to the surface of food products, typically derived from natural, and edible materials. As a functional technology, edible coatings, such as sodium carboxymethyl cellulose (CMC-Na) and sodium alginate (SA), exhibit significant advantages in the preservation of agricultural products [26,27]. By forming a physical barrier, these coatings effectively inhibit microbial invasion and reduce spoilage during the drying process, thereby extending the shelf life of the product [28]. Furthermore, edible coatings can regulate moisture migration, preventing surface hardening and localized excessive water loss which facilitates uniform moisture evaporation and helps maintain the texture and structural integrity of the product [29]. Importantly, edible coatings do not adversely affect the appearance, flavor, or nutritional composition of fruits and vegetables, nor do they pose risks to human health or the environment [30,31]. Compared to traditional chemical pretreatment, edible coating technology better aligns with the current demands of the food industry emphasis on environmental friendliness and safety. Consequently, edible coatings demonstrate considerable potential in the processing of high-value agricultural products, excelling in preserving product quality, optimizing energy efficiency, and extending shelf life.

Until now, despite the significant potential of segmented combination drying in enhancing drying efficiency and improving the quality of agricultural products, research on the effects of MUSVFIR-RFV segmented combination drying on the drying characteristics, physicochemical properties, and sensory attributes of cherries remains unexplored. Especially, studies on the synergistic effects of edible coatings as a pretreatment in conjunction with multi-frequency ultrasonic combined drying are still lacking. Therefore, this study aims to systematically evaluate the comprehensive effects of edible coating pretreatment combined with MUSVFIR-RFV drying on the drying process, physicochemical properties, and sensory evaluation of cherries, thereby providing scientific evidence for the development of energy-efficient, eco-friendly fruit and vegetable drying technologies.

## 2 Materials and methods

### 2.1 Experimental materials

**Fig. 1:**
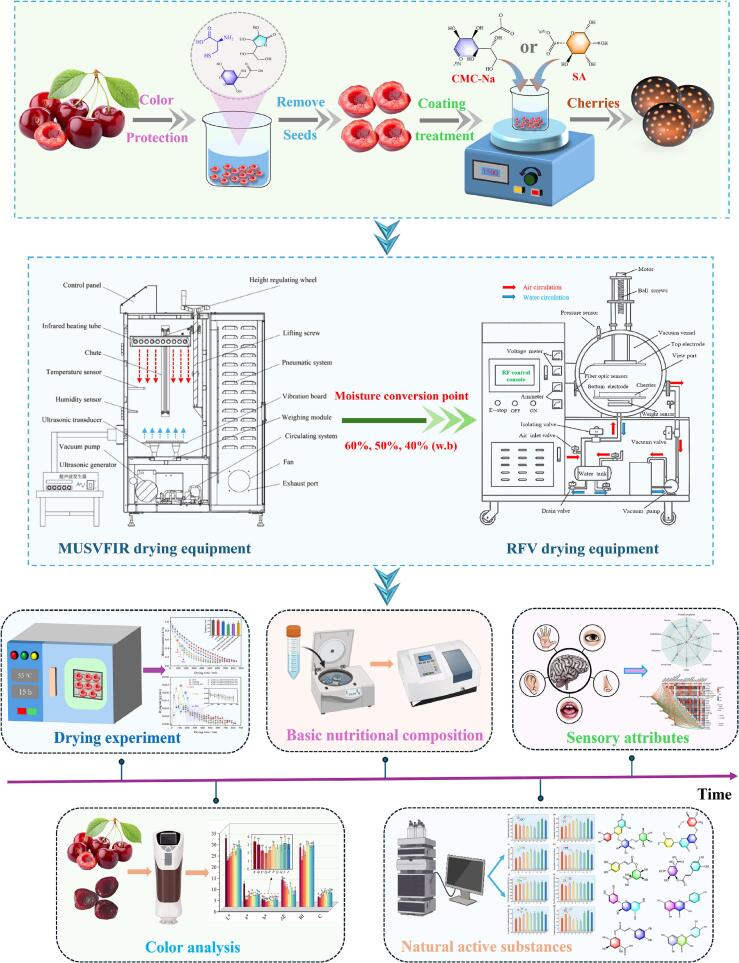
Schematic overview of the experimental procedure.

Fresh cherries with uniform size, maturity, and color were obtained from Tianshui during the peak harvest period. The fruits were carefully inspected to ensure they were free from physical damage, pests, or diseases. To standardize the experimental conditions, the pedicels and seeds were removed, retaining only the flesh for further processing. The initial moisture content was measured at 86.97 ± 1.0 % (w.b) using a moisture detector. To effectively reduce the browning of cherry color and the degradation of anthocyanins, a composite color-preserving solution was prepared, consisting of 0.5 % citric acid, 0.5 % ascorbic acid, and 0.5 % L-cysteine. The cherries (200 g) were immersed in this solution for 10 min and subsequently set aside for further processing. The overall experimental setup is illustrated in Fig. 1.

### 2.2 Control experiments

Radio frequency vacuum drying (RFV): A 200.0 ± 0.5 g sample of fresh cherries was uniformly spread in a single layer on a polypropylene tray and subsequently placed in a RFV drying equipment (GJS-3–27-JY, Hebei Huashi Jiyuan High Frequency Equipment Co., Ltd. Hebei, China) for dehydration. The experimental parameters for RFV drying were set as follows: electrode gap of 90 mm, vacuum pressure of 0.035 MPa, and drying temperature of 55 °C. To prevent localized overheating caused by contact between adjacent samples during RF heating, the cherries were arranged to ensure avoid any overlap or contact. Every 30 min, the sample tray was removed, and the cherries were weighed using an electronic balance with an accuracy of ± 0.01 g. Each weighing process was completed within 20 s to minimize interruption, after which the samples were returned to the drying chamber for continued processing. The experiment was terminated when the moisture content of the cherries decreased to below 15 % (w.b).

Ultrasonic vacuum far infrared radiation drying (USVFIR): Fresh cherries were evenly distributed in a single layer on a stainless steel tray and subsequently placed in an ultrasonic vacuum far-infrared (USVFIR) drying apparatus (KM-615B, Kemeng Ultrasonic Instruments Co., Ltd. Zhejiang, China; WSH-60A infrared radiation drying equipment, Shenghua Microwave Technology Co., Ltd, Tianshui, China). Based on the preliminary experimental studies [32], the experimental conditions for USVFIR drying were set as follows: a drying temperature of 55 °C, an irradiation height of 300 mm, an ultrasonic power of 60 W, and an ultrasonic frequency of 40 kHz.

USVFIR-RFV segmented drying: The drying process of USVFIR-RFV can be divided into two stages. In the first stage, fresh cherry samples were uniformly spread in a single layer on stainless steel trays and subjected to drying using an ultrasonic vacuum far-infrared (USVFIR) system. When the moisture content of the samples decreased to 50 % (w.b), the process wased transferred to the RFV drying stage, continuing until the target moisture content was achieved. Throughout the experiment, the specific drying parameters for each stage were maintained consistent with the predefined settings of the USVFIR and RFV systems to ensure the comparability and reliability of the experimental results.

### 2.3 Coating pretreatment combined with segmented drying experiments

#### 2.3.1 Coating pretreatment

Appropriate amounts of sodium carboxymethyl cellulose (CMC-Na) and sodium alginate (SA) coating materials were weighed and placed into separate beakers. A specified quantity of ultrapure water was added to each beaker, and the mixtures were heated in a water bath at 65 °C while being stirred continuously until fully dissolved, forming a homogeneous and transparent solution. Upon completion of solution preparation, the mixtures were cooled naturally to room temperature. Fresh cherries were then completely immersed in the coating solution for 5 min, after which they were removed and placed on trays to drain excess solution from the surface. The coated cherries were then left at room temperature for 10 min to fix the coating layer.

#### 2.3.2 Segmented combined drying experiments

(CMC-Na)-(MUSVFIR-RFV) segmented combined drying: The (CMC-Na)-(MUSVFIR-RFV) combined drying process was divided into three stages. First, the fresh cherry samples pretreated with CMC-Na coating were evenly spread in a single layer in a stainless steel tray and placed into the ultrasonic vacuum far infrared drying (MUSVFIR) equipment for initial drying. When the moisture content of the samples decreased to 60 %, 50 %, and 40 % (moisture conversion point, w.b), they were sequentially transferred to the RFV drying stage to complete the drying process. Notably, the parameters for the RFV stage remained constant, while in the MUSVFIR stage, the ultrasonic frequency was adjusted to operate in a combined mode of 20 kHz + 40 kHz.

SA-(MUSVFIR-RFV) segmented combined drying: The SA-(MUSVFIR-RFV) combined drying also consists of three stages. The primary difference from the (CMC-Na)-(MUSVFIR-RFV) drying was the substitution of the coating pretreatment material with SA, while the remaining drying procedures and parameter settings were identical.

### 2.4 Moisture content and drying rate

The wet basis moisture content (*MC*) and drying rate (*DR*) were calculated according to the Eq. (1), (2) [33]:

(1) $$\mathrm{BI}=100\times \left[\left(a^{*}+1.75\cdot L^{*}\right)/\left(5.645\cdot L^{*}+a^{*}-3.012\cdot b^{*}\right)-0.31\right]/0.17$$

(2) $$\mathrm{DR}=\left(M_{t}-M_{t-\Delta t}\right)/\Delta t$$

where *M*_t_ is the dry base moisture content at a specific time, g/g; *M*_d_ is the absolutely dried sample, g/g; *M*_t_ and *M_t-_*_Δt_ are the moisture contents on dry basis at time t and t-Δt, respectively, g/g.

### 2.5 Determination of color

The color of both fresh and dried cherries was evaluated using a digital colorimeter (CR-10, Konica Minolta Co., Ltd., Tokyo, Japan) in accordance with the CIELAB color system. Specifically, during the measurements, the D65 standard illuminant was used as the light source, and a 10° standard observer angle was employed to ensure the accuracy and comparability of the results. Color measurements were conducted at three distinct points on each sample, with the mean values of L*, a*, and b* recorded. Each measurement was performed in triplicate to enhance reliability. The total color difference (Δ*E*), browning index (BI), and chroma (C) relative to the fresh sample were calculated using the following Eq. (3), (4), (5) [34,35]:

(3) $$\Delta E=\sqrt{{\left(L^{*}-L_{0}\right)}^{2}+{\left(a^{*}-a_{0}\right)}^{2}+{\left(b^{*}-b_{0}\right)}^{2}}$$

(4) $$\mathrm{BI}=100\times \left[\left(a^{*}+1.75\cdot L^{*}\right)/\left(5.645\cdot L^{*}+a^{*}-3.012\cdot b^{*}\right)-0.31\right]/0.17$$

(5) $$C=\sqrt{{\left(a^{*}\right)}^{2}+{\left(b^{*}\right)}^{2}}$$

where L*, a*, b* represent the brightness/darkness, redness/greenness, yellowness/blueness of dried cherries, whereas L_0_, *a*_0_, *b*_0_ represent the same for fresh cherries.

### 2.6 Analysis of individual sugar, organic acids and natural active substances content

The individual sugars, organic acids, and natural bioactive compounds in dried cherries were quantified using HPLC (Agilent 1100, Agilent Technology Co., Ltd, USA). Prior to analysis, the cherries were ground into a fine powder and extracted with 70 % methanol. The extract underwent centrifugation (4 °C, 12000 rpm) and ultrasonic treatment (200 W, 60 kHz, 25 °C) to ensure thorough extraction. The resulting supernatant was filtered through a 0.45 µm membrane filter prior to injection into the HPLC system. The contents of individual sugars, organic acids, and natural bioactive compounds in cherries were determined following the methods and procedures described by Zang et al [4].

### 2.7 Analysis of total soluble solid

The total soluble solid (TSS) content of dried cherry samples was determined using a PAL-102S digital refractometer (ATAGO Scientific Instrument Co., Ltd, Tokyo, Japan) [36]. Approximately 5 g of dried cherry was finely ground and homogenized. A small portion of the sample extract was obtained by mixing it with distilled water (1:10, w/v), followed by gentle stirring for 10 min. The mixture was then filtered through a 0.45 µm filter to remove solid particles. The refractive index of the filtrate was measured at 25°C. All measurements were performed in triplicate to ensure accuracy and reproducibility.

### 2.8 Analysis of textural property

The texture properties of dried cherries were determined using a texture analyzer (Ta.XT 2i/50, Stable Micro Systems Ltd., United Kingdom). The dried samples were cut into uniform cubes (10 mm × 10 mm × 10 mm), and tested at room temperature using a P/36R cylindrical probe. The testing conditions were set as follows: pre-test speed of 2 mm/s, test speed of 1 mm/s, post-test relaxation speed of 2 mm/s, trigger force of 0.1 N, and a compression ratio of 60 % [37,38]. During the testing process, key texture parameters such as Hardness, springiness, cohesiveness, chewiness, resilience, and gumminess were measured. For each experimental group, five cherries were selected, with each fruit measured at three different positions. The average value of the measurements was used as the final experimental result.

### 2.9 Analysis of total phenolic and total flavonoid contents

The total flavonoid content (TFC) was quantified using the aluminum chloride colorimetric method. In this assay, 10 µL mL of cherries extract were mixed with 2.0 mL of distilled water, 0.3 mL of 5 % NaNO_2_, and 0.3 mL of 10 % AlCl_3_. After 10 min, the absorbance was measured at 510 nm. TFC was expressed as quercetin equivalent (QE) per gram of dry weight [39,40].

The total phenolic content (TPC) was measured using the Folin-Ciocalteu method. Briefly, 0.5 g of dried cherry powder was extracted with 75 % methanol, and 150 µL of the extract was mixed with 2 mL of 10 % Folin-Ciocalteu reagent and 1 mL of 7.5 % sodium carbonate solution. After 30 min of incubation, absorbance was read at 765 nm. Results were expressed as gallic acid equivalents (GAE) per gram of sample [41].

### 2.10 Determination of antioxidant activity

#### 2.10.1 Analysis of DPPH free radical scavenging activity

The DPPH radical scavenging activity of dried cherries were evaluated following the method described by Zang et al. [4,24], with slight modifications where necessary. The assay was conducted using a spectrophotometer to measure the absorbance at 517 nm, reflecting the antioxidant capacity of the cherry through its ability to neutralize DPPH radicals.

#### 2.10.2 Analysis of ABTS reducing antioxidant power

The ABTS solution was prepared by mixing an appropriate amount of ABTS with phosphate-buffered saline (pH 7.4) and hydrogen peroxide. The mixture was incubated at room temperature for 60 min until the absorbance stabilized within the range of 0.7 to 0.8. Subsequently, 40 μL of the sample was thoroughly mixed with 3 mL of the prepared ABTS solution and allowed to react at room temperature for 10 min. The absorbance was then recorded at 734 nm. The ABTS^+^ radical scavenging activity of the sample was quantified and expressed as Trolox equivalent antioxidant capacity [42,43].

#### 2.10.3 Analysis of ferric ion reducing antioxidant power (FRAP)

The FRAP assay was performed based on the method described by Lopes et al. [44] and Yang et al. [45], with minor modifications to suit the experimental requirements. Specifically, 20 μL of the sample extract was thoroughly mixed with 2 mL of 500 μmol·L^–1^ Trolox standard solution and 6 mL of FRAP reagent. The mixture was incubated at 37 °C in a water bath for 30 min, followed by absorbance measurement at 593 nm to determine the antioxidant capacity of the cherry.

### 2.11 Analysis of sensory attributes

Sensory attributes were evaluated by a trained panel of 20 experts from Gansu Agricultural University, specializing in fruit sensory analysis. A standardized sensory evaluation protocol was followed, assessing key attributes using a 10-point hedonic scale. Prior to evaluation, panelists underwent training to standardize sensory descriptors and evaluation criteria. All assessments were conducted under controlled environmental conditions to minimize external variability. Each sample was evaluated in triplicate, and results were expressed as the mean score for each attribute [46].

### 2.12 Statistical analysis

Data were analyzed using SPSS 25.0 Statistics software (IBM Corp., Armonk, NY, USA), Origin 2022 software (OriginLab Corporation., Massachusetts, USA), and Microsoft Excel 2010 (Microsoft Corporation., Washington, USA). All results are expressed as the mean ± standard deviation of three independent replicates. Statistical comparisons between groups were performed using one-way analysis of variance (ANOVA), followed by Tukey’s HSD test to assess significant differences.

## 3 Results and discussion

### 3.1 Drying characteristics

**Fig. 2:**
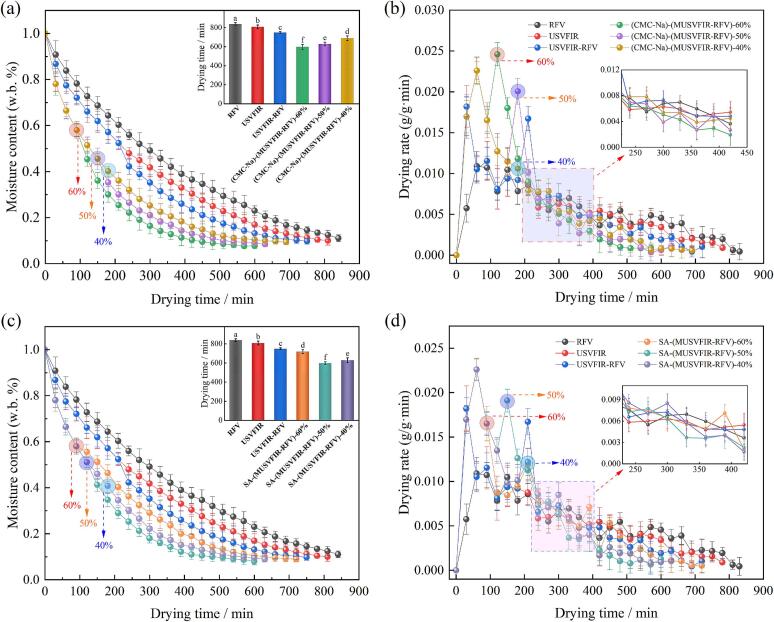
Drying characteristic curves of cherries under various drying conditions. ((a,c). Moisture content (w.b) after CMC-Na or SA pretreatment, (b,d). Drying rate following CMC-Na or SA pretreatment).

The drying characteristic curves of cherries under different dehydration treatments are shown in Fig. 2. The drying time of cherries subjected to USVFIR-RFV treatment was 720 min, which was reduced by 90 min and 60 min compared to RFV and USVFIR treatments, respectively. Meanwhile, the drying rate of cherries after USVFIR-RFV treatment reached 0.65 g/g·min, representing an increase of 22.64 % and 12.07 % compared to RFV and USVFIR, respectively. This indicated that compared to single drying, segmented drying technology could reasonably regulate temperature and energy distribution at different stages, thereby avoiding overheating and uneven moisture distribution, significantly improving drying efficiency. Specifically, during the initial drying stage, USVFIR drying could effectively enhance heat and mass transfer rates as well as moisture migration. Additionally, VFIR heating facilitated rapid water removal at relatively low temperatures. Simultaneously, the cavitation and mechanical vibration effects of ultrasound promoted cell wall permeability and weakened cohesion between molecules, thereby enabling easier diffusion of water from the interior of the cherries to the surface [19,22,23].

Compared to the USVFIR-RFV treatment, cherries pretreated with CMC-Na and SA coatings followed by MUSVFIR-RFV segmented drying exhibited a 4.17 % to 16.7 % reduction in drying time and a 5.56 % to 46.14 % increase in drying rate. This is because the different frequencies of multi-frequency ultrasound act at different stages and depths of drying, creating a synergistic effect. For instance, low-frequency ultrasound can accelerate the evaporation of surface moisture during the initial stage through cavitation and vibration effects, while high-frequency ultrasound facilitated the migration of moisture from deeper layers [47]. Consequently, the synergistic effect of multi-frequency bands results in a more uniform drying process, avoiding the phenomenon of surface over-drying and internal moisture retention in single-frequency ultrasonic drying, thereby enhancing the drying rate [48,49]. Furthermore, although the application of coating technology may impede the increase in evaporation rate during the cherry dehydration process, the coating prevented rapid surface drying that could lead to skin cracking or uneven moisture evaporation within the fruit. Under the combined effect of multi-frequency ultrasound and radio frequency, the barrier effect of the coating was disrupted, thus accelerating moisture migration and evaporation. Subsequent research results also indicated that edible coatings not only improved drying performance but also effectively maintained the color, flavor, and nutritional components of cherries. Similar conclusions have been reached by Zang et al [4]. Notably, after CMC-Na pretreatment, the drying time (600 min) was the shortest and the drying rate (0.95 g/g·min) was the highest when the moisture conversion point reached 60 %; whereas after SA treatment, the shortest dehydration time was observed when the moisture conversion point was 50 %.

### 3.2 Color

Color is a critical quality attribute of dried fruits, significantly influencing consumers' visual perception, assessment of nutritional value, and purchase intention [50]. Table 1 presents the effects of different drying methods on the color of cherries. Fresh samples exhibited a relatively high brightness level, with an *L*^∗^ value of 30.59. In contrast, the brightness of samples dried using RFV (*L*^∗^=20.77) and USVFIR (*L*^∗^=21.91) was markedly reduced. This reduction is primarily attributed to non-enzymatic browning induced by polyphenol oxidase (PPO) activity during prolonged high-temperature drying, as well as intensified Maillard reactions, which further darkened the color. Compared to the RFV and USVFIR methods, the brightness of samples subjected to the USVFIR-RFV sequential drying process was slightly improved (*L*^∗^=22.67), suggesting that the combined sequential drying approach could effectively mitigate browning to some extent. Moreover, the samples treated with edible coatings exhibited consistently higher brightness values, particularly those coated with CMC-Na, which had brightness values ranging from 25.48 to 27.84. This suggested that the coating acted as an effective oxygen barrier, thereby inhibiting enzymatic browning [51]. As shown in Table 1, all drying treatments resulted in a significant reduction in the *a*^∗^ values of cherries, with the most pronounced decreases observed under RFV and USVFIR drying conditions, where the *a*^∗^ values dropped to 3.17 and 3.08, respectively. This reduction was likely attributed to the degradation of anthocyanins and the intensification of browning reactions during the drying process, leading to a diminished red hue. Further analysis revealed that the Cyanidin-3-O-rutinoside content in cherries dried using RFV and USVFIR was 18.49 ± 0.97 and 19.33 ± 1.05 mg/100 g, respectively, which was significantly lower than the corresponding values for samples dried using USVFIR-RFV, SA-(MUSVFIR-RFV), and (CMC-Na)-(MUSVFIR-RFV). The reductions were quantified as 24.99 %, 27.46 ∼ 31.67 %, 25.29 ∼ 36.63 %, and 21.58 %, 24.17 ∼ 28.57 %, 21.90 ∼ 33.76 %, respectively (Fig. 4 (g)). These findings further confirmed a positive correlation between anthocyanin content and *a*^∗^ values, indicated that preserving anthocyanins could mitigate the reduction in red hue during the drying process. Notably, samples pretreated with edible coatings and subjected to MUSVFIR-RFV segmented drying demonstrated superior retention of red hues. This improvement was likely attributed to the protective barrier provided by the coating, which effectively reduced the degradation of thermosensitive pigments and flavonoids. Furthermore, functional components in the coating might mitigate oxidative damage through antioxidant mechanisms, thereby enhanced color stability. The total color difference (Δ*E*) reflected the degree of color deviation between dried products and fresh cherries. Dried cherries processed with (CMC-Na)-(MUSVFIR-RFV)-50 % exhibited the smallest Δ*E* value (5.16 ± 0.68) and the highest chroma value (C = 6.69 ± 0.31). This indicated that when moisture conversion point was 50 %, MUSVFIR-RFV segmented drying effectively optimizes temperature and energy distribution, thereby minimizing the degradation of pigments such as anthocyanins and carotenoids under high-temperature and humid conditions, ultimately preserved the product's color quality [52].

**Table 1:** Color parameters and total soluble solid content of cherries under different drying conditions.

Drying conditions	L*	a*	b*	Δ*E*	BI	C	TSS
Fresh	30.59 ± 2.41^a^	10.24 ± 0.27^a^	3.41 ± 0.12^a^	—	—	—	14.75 ± 0.47^a^
RFV	20.77 ± 2.02^f^	3.17 ± 0.42^d^	3.03 ± 0.19^bc^	12.10 ± 1.57^a^	26.52 ± 2.04^ab^	4.39 ± 0.15^d^	9.49 ± 0.34^e^
USVFIR	21.91 ± 1.97^e^	3.08 ± 0.37^d^	2.28 ± 0.08^cd^	11.28 ± 1.68^ab^	20.88 ± 1.59^d^	3.83 ± 0.22^e^	9.44 ± 0.27^e^
USVFIR- RFV	22.67 ± 1.36^e^	5.34 ± 0.33^bc^	1.99 ± 0.18^d^	9.38 ± 1.06^b^	25.61 ± 1.83^b^	5.70 ± 0.20^b^	9.45 ± 0.25^e^
SA- (MUSVFIR- RFV)-60 %	24.33 ± 2.41^d^	4.55 ± 0.19^cd^	2.32 ± 0.21^cd^	8.50 ± 1.27^c^	23.14 ± 1.37^c^	5.11 ± 0.18^cd^	10.45 ± 0.33^de^
SA- (MUSVFIR- RFV)-50 %	26.42 ± 1.54^bc^	5.91 ± 0.32^b^	3.04 ± 0.19^bc^	6.01 ± 0.94^e^	27.89 ± 2.01^a^	6.65 ± 0.16^a^	11.28 ± 0.41^d^
SA- (MUSVFIR- RFV)-40 %	23.09 ± 2.07^de^	5.03 ± 0.22^c^	2.67 ± 0.15^c^	9.14 ± 0.68^b^	27.56 ± 2.33^a^	5.69 ± 0.20^c^	13.14 ± 0.29^bc^
(CMC-Na)-(MUSVFIR- RFV)-60 %	26.04 ± 1.96^bc^	4.98 ± 0.31^c^	3.01 ± 0.23^bc^	6.96 ± 0.77^d^	25.72 ± 1.82^b^	5.82 ± 0.14^b^	12.29 ± 0.40^c^
(CMC-Na)- (MUSVFIR- RFV)-50 %	27.84 ± 1.49^b^	5.88 ± 0.27^b^	3.19 ± 0.20^b^	5.16 ± 0.68^f^	26.98 ± 1.47^ab^	6.69 ± 0.31^a^	12.32 ± 0.39^c^
(CMC-Na)- (MUSVFIR- RFV)-40 %	25.48 ± 2.01^c^	5.37 ± 0.24^bc^	3.08 ± 0.16^bc^	7.06 ± 0.61^d^	27.67 ± 2.18^a^	6.19 ± 0.23^ab^	13.48 ± 0.26^b^

Note: Data are expressed as means ± standard deviation of triplicate samples. Values with different letters represent significant difference at the level of *P* < 0.05. (The same below).

### 3.3 Individual sugars and organic acids

**Fig. 3:**
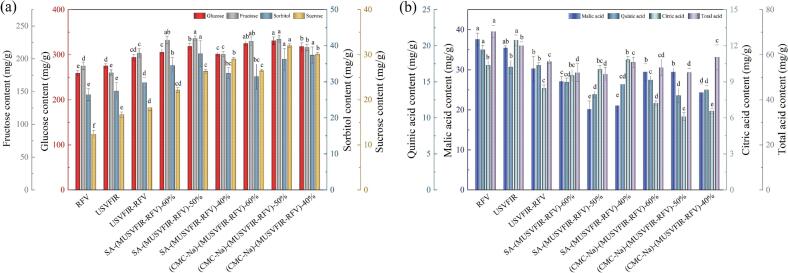
Individual sugars (a) and organic acids (b) contents in cherries under different drying processes.

Individual sugars and organic acids are not only key nutritional components of cherries but also play significant roles in sensory attributes such as flavor, color, and texture, collectively determining the physicochemical quality of these products [53]. Fig. 3 demonstrated the changes in carbohydrate and acid content in dried cherries following different dehydration treatments. The study found that compared to USVFIR-RFV treatments, the contents of glucose, fructose, sorbitol, and sucrose in cherries increased by 2.38 ∼ 12.40 %, 0.78 ∼ 11.46 %, 5.61 ∼ 26.96 % and 21.55 ∼ 75.55 % after drying with SA-(MUSVFIR-RFV) and (CMC-Na)-(MUSVFIR-RFV), respectively. This was attributed to the excellent water solubility and adhesiveness of CMC-Na and SA, which formed a stable protective film on the surface of cherries, reduced excessive concentration of sugars and preventing rapid water loss. This helped avoid caramelization, denaturation, and internal thermal damage to sugars, thus slowing their degradation. Notably, a higher concentration of sugars was observed in the (CMC-Na)-(MUSVFIR-RFV) treated samples. SA could form crosslinked structures with metal ions, leading to the formation of reversible gels, which enhanced the mechanical strength of the film but also inhibited water evaporation to some extent, and reduced drying efficiency. This results in cherries being exposed to a prolonged high-temperature and high-humidity environment, which facilitated the thermal degradation of glucose and sucrose. This suggested that CMC-Na better preserved the carbohydrate content in cherries than SA. Additionally, compared to single-frequency treatments (USVFIR and USVFIR-RFV), multi-frequency ultrasound significantly increased the carbohydrate content in cherries (*P* < 0.05). This could be attributed to multi-frequency ultrasound improving the uniformity of heat distribution during drying, ensuring even heating of both the surface and interior of the cherries. Moreover, the unique cavitation effect of multi-frequency ultrasound accelerated water evaporation, enhanced cell wall permeability, and facilitated the release of soluble substances, which helped to increase the concentration, flavor, and nutritional value of sugars in dried cherries [54]. After dehydration with (CMC-Na)-(MUSFIR-RFV)-50 % and SA-(MUSFIR-RFV)-50 %, the highest contents of glucose and fructose were found to be 174.81 ± 3.33 mg/g, 165.43 ± 4.12 mg/g and 12.37 ± 0.45 mg/g, 11.92 ± 0.37 mg/g, respectively. As the moisture conversion point decreased from 60 % to 40 %, the sugar content first increased and then decreased. At a moisture conversion point of 60 %, the drying rate during the RFV stage was slower, leading to a longer dehydration time, which increased the likelihood of sugar degradation. When the conversion water content was 40 %, prolonged ultrasound exposure caused cell wall rupture, promoted the release and diffusion of sugars, but also resulted in partial hydrolysis of the sugars.

Malic acid (37.58 ± 1.12 mg/g), quinic acid (19.47 ± 0.84 mg/g), and total acid content (70.33 ± 1.56 mg/g) were observed to be the highest in the cherries pretreated with RFV, which were 1.27, 1.49, 1.35 and 1.87, 1.47, 1.37 times higher than that of the (CMC-Na)-(MUSFIR-RFV)-50 % and SA-(MUSFIR-RFV)-50 %, respectively. The highest citric acid content was observed in cherries dried using USVFIR. Cherries pretreated with CMC-Na and SA edible coatings exhibited a slight reduction in acidity. On the one hand, while the edible coatings provided some protection to the cherry surface, slowing down moisture loss and oxidation, they cannot completely prevented the loss of heat-sensitive compounds, such as internal acids, during the thermal treatment process. On the other hand, multi-frequency ultrasound energy can induce cavitation effects that disrupt cell walls, released cell sap and intracellular components, potentially caused cellular damage and chemical reactions that lead to the loss of certain acidic compounds. RF treatment might exacerbate the reaction between acids and sugars or other organic compounds, resulting in the formation of non-acidic substances and further contributing to the degradation or transformation of acidic compounds.

### 3.4 Natural active substances

**Fig. 4:**
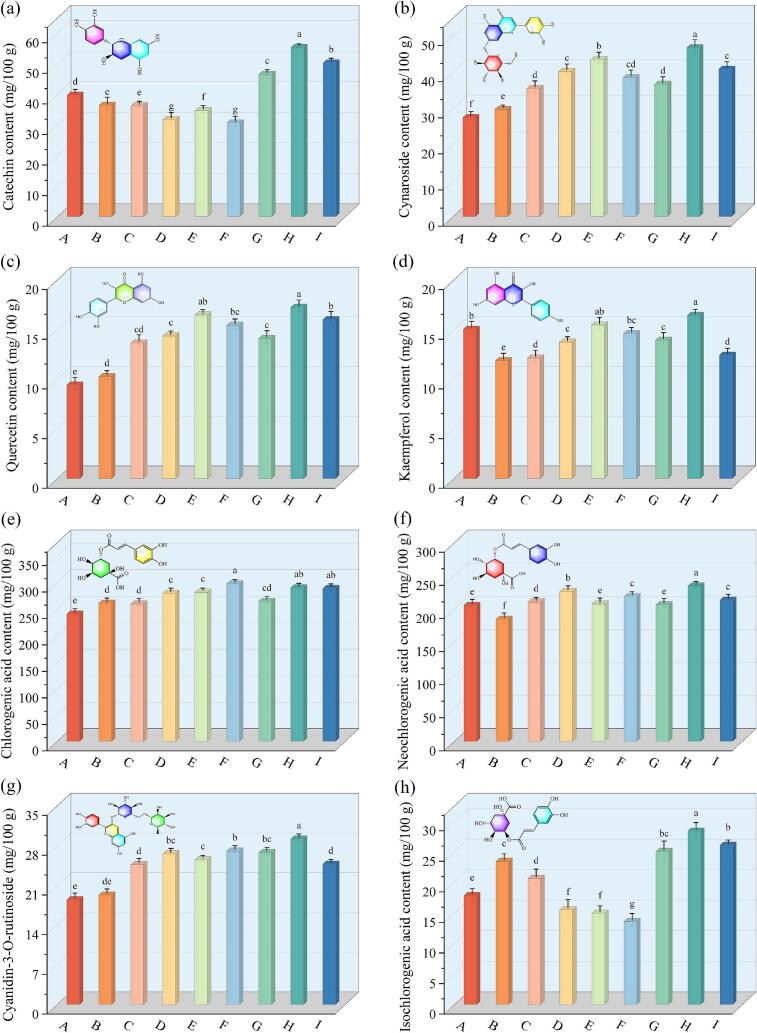
Content of various natural active substances in cherries under various drying conditions.

HPLC was utilized to determine the content of catechin, chlorogenic acid, neochlorogenic acid, isochlorogenic acid, cynaroside, quercetin, kaempferol, and cyanidin-3-O-rutinoside components in RFV, USVFIR, USVFIR-RFV, and MUSVFIR-RFV dried cherries pretreated with CMC-Na and SA edible coating (Fig. 4). The concentrations of catechin, chlorogenic acid, and neochlorogenic acid in dried cherries across various treatments ranged from 31.77 ± 1.37 to 55.38 ± 2.37, 240.59 ± 4.26 to 297.58 ± 6.01, and 184.55 ± 3.94 to 234.59 ± 3.07 mg/100 g, respectively. These compounds were critical natural bioactive constituents contributing to the flavor profile of cherries. After dehydration using USVFIR-RFV, SA-(MUSFIR-RFV)-60 %, SA-(MUSFIR-RFV)-40 %, (CMC-Na)-(MUSFIR-RFV)-60 %, and (CMC-Na)-(MUSFIR-RFV)-40 %, the kaempferol content in cherries was 12.15 ± 0.42, 13.75 ± 0.33, 14.59 ± 0.29, 13.93 ± 0.24, and 12.44 ± 0.38 mg/100 g, respectively, representing reductions of 19.27 %, 8.64 %, 3.06 %, 7.44 %, and 17.34 % compared to the RFV treatment. These results suggested that RFV technology offered significant advantages in preserving kaempferol content. This could be attributed to the direct interaction of RF heating with the internal molecular structure of the material through electromagnetic waves. In contrast to conventional thermal convection and conduction methods, RF heating offered superior penetration and uniformity, which more effectively preserved heat-sensitive compounds and minimized nutrient loss due to overheating of the outer layer [55]. Isochlorogenic acid, neochlorogenic acid, kaempferol, quercetin, and cynaroside contents were highest in cherries treated with (CMC-Na)-(MUSFIR-RFV)-50 %, with values 1.90, 1.13, 1.06, 1.05, and 1.08 times higher than those of SA-(MUSFIR-RFV)-50 %, respectively. CMC-Na, a water-soluble polysaccharide with strong hydration properties, effectively mitigated excessive dehydration damage to fruit tissues by forming a protective film on the cherry surface, thereby preserving the natural nutrients and flavor compounds. Although SA coatings also inhibited the degradation of bioactive components, they were more prone to hygroscopicity in humid conditions, which reduced the stability of the coating and diminished its protective effects. Additionally, the data in Fig. 4 showed that multi-frequency ultrasound provided significant benefits in preserving the natural active compounds of cherries, suggesting its potential applicability. For instance, neochlorogenic acid and catechin contents in USVFIR-dried cherries were reduced by 10.18 % and 9.17 %, respectively, compared to RFV-dried cherries. However, the content of most natural active substances increased significantly after treatment with MUSVFIR. Overall, the combined application of edible coating pretreatment and MUSFIR-RFV segmented drying achieved a synergistic effect, enhancing drying efficiency, minimizing thermal damage and oxidative degradation during the drying process, and maximizing the retention and enhancement of natural bioactive compounds in cherries..

### 3.5 Soluble solid

Soluble solids content (TSS) is a critical indicator for assessing the levels of sugars, organic acids, and other water-soluble compounds in fruits [56]. Experimental results revealed that the TSS in cherries subjected to RFV, USVFIR, and USVFIR-RFV drying treatments were 9.45, 9.44, and 9.49 °Bx, respectively (Table 1), indicated that segmented drying without coating pretreatment had no significant effect on enhancing TSS (*P* > 0.05). This phenomenon may be attributed to the rapid heating effect during the RFV stage, which facilitated uniform dehydration but had limited influence on the concentration of soluble compounds. The combination of coating pretreatment and MUSVFIR-RFV segmented drying significantly increased TSS content (*P* < 0.05). Notably, cherries treated with (CMC-Na)-(MUSVFIR-RFV)-40 % exhibited the highest TSS content of 13.48 °Bx. This improvement could be explained by the semi-permeable membrane formed by the CMC-Na coating during drying, which effectively reduced the evaporation rate while limiting the migration loss of soluble small molecules, such as sugars and organic acids. Correlation analysis further supported this finding, showing a significant positive correlation between TSS content and sugars as well as organic acids. Additionally, multi-frequency ultrasound treatment likely enhanced the diffusion and redistribution of soluble solids within the fruit, thereby contributing to a substantial increase in TSS after drying.

### 3.6 Texture

Hardness, springiness, cohesiveness, chewiness, resilience, and gumminess are key texture indicators in cherry drying, which not only provided a comprehensive evaluation of the drying process's impact on the texture and sensory quality of dried fruit products but also offered a scientific foundation for optimizing drying procedures and improved product quality [57]. The results of textural properties in cherries subjected to various dehydration treatments are presented in Table 2. Compared to single RFV and USVFIR drying, the resilience, chewiness, and cohesiveness of dried cherries significantly increased, whereas hardness and gumminess exhibited a decreasing trend after combined pretreatment and segmented drying. CMC-Na and SA coatings effectively mitigated rapid water migration during the early drying stage by regulating moisture retention and heat conduction, thereby reducing the shrinkage and hardening of the cell wall caused by rapid water loss and high temperatures. This resulted in a decrease in the overall hardness of the cherries. Additionally, multi-frequency ultrasound alleviated surface hardening by promoting uniform thermal distribution. The cavitation bubble effect also enhanced the permeability of the cell membrane, weakened the structural integrity of the cell wall, and softened the pulp tissue, further reducing hardness. Furthermore, the cavitation effect of ultrasound disrupted the cell wall and intercellular substance, loosening the fruit tissue and weakening intercellular bonding, which ultimately led to a reduction in gumminess [58].

**Table 2:** Textural properties of cherries under different drying processes.

Drying conditions	Hardness (N)	Springiness (%)	Cohesiveness	Chewiness (N)	Resilience (%)	Gumminess (N)
RFV	21.08 ± 0.53^a^	58.49 ± 2.79^f^	0.34 ± 0.07^e^	5.78 ± 0.18^cd^	25.48 ± 2.33^d^	8.41 ± 0.10^a^
USVFIR	18.44 ± 0.37^b^	62.07 ± 3.01^e^	0.31 ± 0.06^e^	6.02 ± 0.21^c^	23.94 ± 1.94^e^	6.37 ± 0.08^c^
USVFIR- RFV	16.37 ± 0.29^c^	67.84 ± 3.48^d^	0.49 ± 0.08^d^	5.34 ± 0.14^d^	30.55 ± 3.05^c^	7.08 ± 0.12^b^
SA- (MUSVFIR- RFV)-60 %	13.49 ± 0.33^de^	72.59 ± 4.06^c^	0.64 ± 0.12^bc^	7.39 ± 0.20^ab^	31.08 ± 2.10^c^	4.37 ± 0.07^e^
SA- (MUSVFIR- RFV)-50 %	12.74 ± 0.21^e^	79.33 ± 2.11^b^	0.72 ± 0.11^b^	6.54 ± 0.09^b^	34.41 ± 2.37^b^	3.81 ± 0.08^f^
SA- (MUSVFIR- RFV)-40 %	15.42 ± 0.35^cd^	66.01 ± 3.05^d^	0.67 ± 0.07^bc^	6.28 ± 0.14^bc^	23.07 ± 2.44^e^	5.67 ± 0.14^d^
(CMC-Na)-(MUSVFIR- RFV)-60 %	14.02 ± 0.24^d^	75.28 ± 2.19^bc^	0.61 ± 0.13^c^	5.91 ± 0.12^c^	30.07 ± 1.69^c^	6.44 ± 0.13^c^
(CMC-Na)- (MUSVFIR- RFV)-50 %	13.44 ± 0.36^de^	83.11 ± 3.97^a^	0.78 ± 0.05^a^	7.82 ± 0.17^a^	36.27 ± 2.06^a^	4.28 ± 0.07^e^
(CMC-Na)- (MUSVFIR- RFV)-40 %	16.08 ± 0.40^c^	78.97 ± 4.01^b^	0.60 ± 0.11^c^	6.28 ± 0.08^bc^	26.55 ± 1.71^d^	5.91 ± 0.11^cd^

The highest values for springiness (83.11 ± 3.91), cohesiveness (0.78 ± 0.14), chewiness (7.82 ± 0.59), and resilience (36.27 ± 2.25) were obtained in cherries treated with (CMC-Na)-(MUSVFIR-RFV)-50 %. These values increased by 10.40 %, 27.89 %, 32.32 %, 20.62 %, and 5.24 %, 30.00 %, 24.52 %, 60.84 % compared to the cherries with 60 % and 40 % moisture conversion point, respectively. When the moisture conversion point was 40 %, prolonged ultrasonic treatment aggravated the degradation of pectin and hemicellulose, increasing the concentration of degradation products and weakening the elasticity of the cell wall, thereby reducing springiness and chewiness [4]. At a moisture conversion point of 60 %, the liquid water between the cells acted as a “lubricant,” reducing the bonding between cell walls due to the high moisture content. Prolonged RFV heating subsequently caused irreversible deformation of the internal cherry structure, leading to a decrease in textural properties. In contrast, at moisture conversion point of 50 %, the water distribution within the cherries was more uniform, the cell walls retained moderate flexibility, and the support provided by the intercellular substances was optimal. Furthermore, the segmented heating by MUSVFIR and RFV promoted uniform heat transfer, prevented the hardening of the cell walls and the reduction of mechanical strength due to sudden water loss, thereby exhibiting the optimal textural properties.

### 3.7 TPC and TFC

**Fig. 5:**
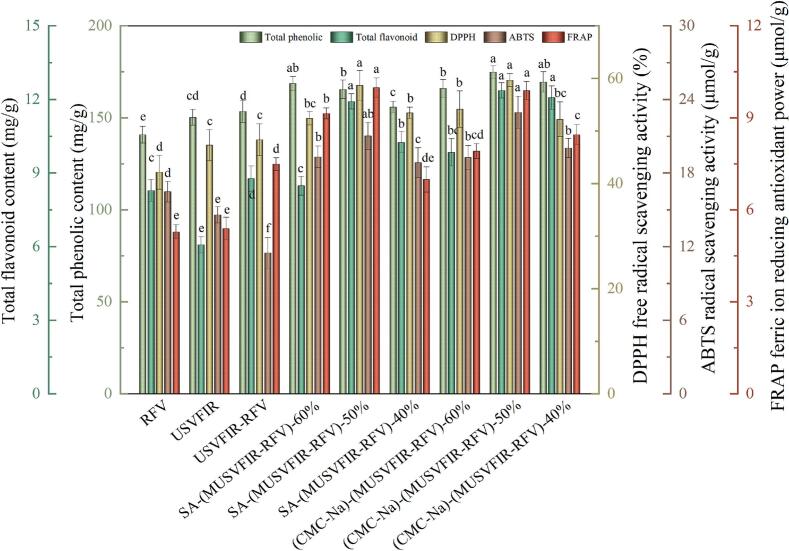
TPC and TFC, and antioxidant capacity (DPPH, ABTS, and FRAP) of cherries under different drying processes.

TPC and TFC are important bioactive components in fruits and vegetables, they not only help to promote the antioxidant capacity and prolong the shelf life, but also improve the nutritional value, sensory quality as well as the market competitiveness [59]. The TPC and TFC of cherries under different dehydration treatments conditions are shown in Fig.5. The results showed that different pretreatment methods had significant effects on the TPC and TFC of cherries (*P* < 0.05). According to Fig. 5, (CMC-Na)-(MUSFIR-RFV)-50 % dried cherry samples had the highest TPC (174.81 ± 4.35 mg/g) and TFC (12.37 ± 0.59 mg/g) and were increased by 13.90 % and 40.05 %, respectively, compared to USFIR-RFV. The SA-(MUSFIR-RFV) treatment group also showed similar conclusions. This indicated that edible coating pretreatment combined with segmental drying inhibited the thermal degradation of phenolic and flavonoid compounds and effectively contributed to the retention of physicochemical qualities and flavor components of cherries. Because the CMC-Na and SA edible coatings effectively isolate oxygen and reduce the oxidative reactions of phenolic and flavonoid compounds, thereby reducing the degradation of these compounds [4,27]. Furthermore, the TPC (153.48 ± 3.68 mg/g) and TFC (8.77 ± 0.43 mg/g) in cherries were increased by 9.02 %, 2.13 % and 5.79 %, 44.48 %, respectively, after segmented combined drying (USVFIR-RFV) compared to single-stage drying methods (RFV and USVFIR). Under the action of ultrasound, the rupture of cell structure and the increase of pores can help the moisture to migrate more easily from the inside to the surface, which effectively shorten the drying cycle, thus reducing the contact time of TPC and TFC with the high-temperature environment [24,60]. Secondly, combined drying can avoid the local overheating and pyrolysis in a single drying, and help to maintain the heat-sensitive compounds in cherries. The results showed that compared with the single drying method, the segmented combined drying could fully utilize the synergistic effect of ultrasound, far-infrared radiation and RFV to ensure the drying efficiency while reducing the damage to phenolic and flavonoid compounds. The TPC and TFC in cherries pretreated with CMC-Na and SA coating ranged from 155.91 ± 4.01 to 174.81 ± 4.37 mg/g and 8.48 ± 0.32 to 12.37 ± 0.45 mg/g, respectively, and showed a significant increase in TPC and TFC compared with RFV, USVFIR and USVFIR-RFV. This might be because the edible coating could isolate oxygen, and the application of multi-frequency ultrasound could make the energy evenly distributed throughout the sample, reducing the generation of local hot spots. This uniform energy distribution helped to maintain a “low-temperature” environment during the drying process, thus avoiding the degradation of phenolics and flavonoids caused by the energy concentration effect.

### 3.8 Antioxidant capacity

Cherries are rich in antioxidant substances such as vitamin C, anthocyanins, flavonoids and polyphenols, which help to prevent oxidative damage to cells and play a variety of health effects including anti-aging and anti-cancer. In recent years, a large number of studies have shown that dried fruit products with strong antioxidant capacity have significant advantages in maintaining their nutritional value, prolonging shelf life and enhancing consumer health [61,62]. Fig. 5 demonstrated the antioxidant capacity (DPPH, ABTS and FRAP) of dried cherries after CMC-Na and SA coating pretreatment and MUSFIR-RFV segmented drying. The USVFIR-RFV, SA-(MUSFIR-RFV) and (CMC-Na)-(MUSFIR-RFV) cherries had DPPH free radical scavenging activity of 48.33 %, 52.14 ∼ 58.67 % and 52.22 ∼ 59.67 %, respectively, which were significantly higher than the RFV (42.14 %) and USVFIR (47.29) cherries. This was because multi-frequency ultrasonic treatment enhanced the cell permeability and cell wall rupture of cherry fruits through high-frequency vibration and promoted the dissolution of antioxidant components (e.g., flavonoids, polyphenols, and anthocyanins) in the samples, which improved the antioxidant capacity of cherries. The ability of multi-frequency ultrasound treatment to significantly enhance the retention of antioxidant components in fruits was also reported in the results of Xu et al [47,49]. Simultaneous application of edible coating pretreatment and segmental combined drying can indirectly or directly promote peroxidase (POD) and superoxide dismutase (SOD) activities. Enhanced antioxidant enzyme activity contributes to increased antioxidant capacity and reduced oxidative reactions in dried cherries. The highest DPPH and ABTS radical scavenging activity were found in cherries at 59.67 %, 22.94 μmol/g (CMC-Na) and 58.67 %, 21.02 μmol/g (SA) when the converted moisture content was 50 %. The ABTS radical scavenging activity was increased by 19.05 %, 14.47 % and 8.86 %, 11.57 % compared to the converted moisture content of 60 % and 40 %, respectively. Interestingly, ABTS radical scavenging activity was increased by 43.55 % and 27.09 % in RFV and USVFIR dried cherries compared to USVFIR-RFV. The above results might be attributed to the mechanical energy and cavitation effect generated by ultrasound causing changes in the local temperature and pH of the solution, which inhibited the activity of antioxidant substances, led to a decrease in ABTS free radical scavenging rate. This also reflected the unique advantages and potential value of CMC-Na and SA coating pretreatment in maintaining antioxidant substances and enhancing antioxidant activity [63]. A maximum FRAP value of 9.98 μmol/g was observed in the SA-(MUSFIR-RFV)-50 % dried cherries, which can be attributed to its high protective and synergistic effects. Moreover, there was a significant positive correlation between antioxidant capacity and TPC and TFC content of cherries.

### 3.9 Sensory evaluation

**Fig. 6:**
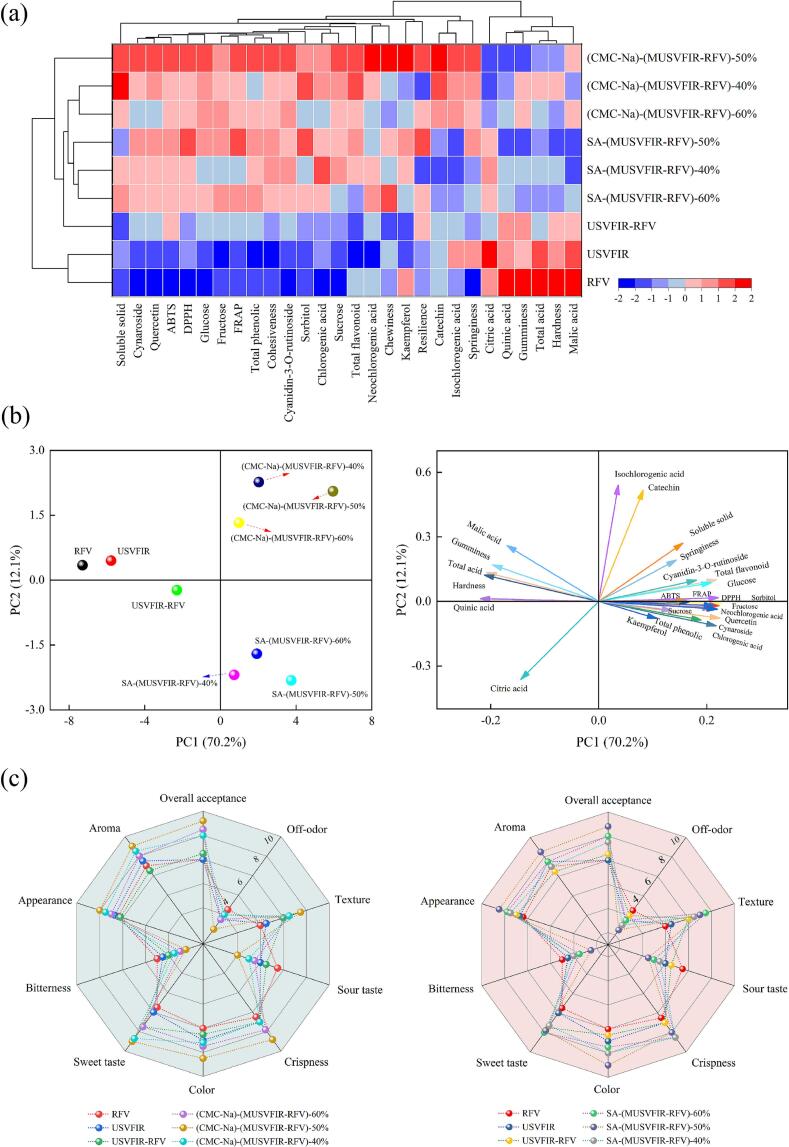
Hierarchical clustering analysis (a), PCA analysis (b), and sensory evaluation (c) in cherries under various drying processes.

Sensory properties are critical indicators for evaluating the physicochemical quality of dried cherry products that sensory evaluation can reflect the consumer's acceptance of the product more intuitively, compared to chemical composition analysis alone. (CMC-Na)-(MUSVFIR-RFV)-50 % and SA-(MUSVFIR-RFV)-50 % treated dried cherry products had relatively high overall acceptance scores of 9.2 and 8.8, respectively (Fig. 6(c)). Dried cherries pretreated with CMC-Na and SA coatings performed better on texture, crispness, color, sweet taste, appearance, and aroma scores than RFV and USVFIR treatments. This might be attributed to the formation of a semipermeable barrier by CMC-Na and SA coatings during the drying process, which effectively slowed the excessive loss of sugars and organic acids, thereby better preserving the endogenous sugar-to-organic acid ratio in cherries. Additionally, the coating reduced color degradation, the formation of surface cracks, and the oxidative damage to flavor compounds [64]. The MUSVFIR-RFV segmented drying technology facilitated uniform heat transfer and moisture evaporation, thereby mitigating issues such as excessive dehydration and uneven drying (e.g., localized carbonization). This process enhanced cell wall elasticity and strength, improved texture and crispness, and effectively reduced the loss of volatile aromatic compounds. Studies have demonstrated that bitterness and off-flavors are undesirable taste components for most consumers. During dried fruit products, excessive bitterness and off-odor could overshadow the natural aroma and flavor of the fruit, significantly reduced the attractiveness of dried fruit. The highest bitterness (3.0) and off-odor (2.5) scores were obtained in RFV-treated dried cherries, which increased 1.20 and 1.67 times compared to the USVFIR treatment. Due to the relatively low drying rate during the RFV drying process, certain compounds in cherries, such as polyphenolic substances (e.g., vitamin C, catechins, and chlorogenic acid), undergo hydrolysis or oxidation under prolonged high-temperature and humid conditions. Furthermore, in single-stage RFV drying, uneven heat distribution might occur within the cherries, leading to localized overheating. This could cause intense thermal reactions involving sugars, amino acids, and organic acids, resulting in the formation of by-products associated with off-flavors and bitterness.

### 3.10 PCA analysis and hierarchical clustering analysis

Fig. 6(a) presents the heatmap analysis and hierarchical clustering results of the physicochemical quality attributes of dried cherry products obtained using different drying methods. The analysis indicated significant compositional differences among the cherries processed with eight distinct drying treatments, which were largely consistent with the results of the principal component analysis (Fig. 6(b)). Samples treated with RFV and USVFIR were clustered into one group, characterized by relatively lower levels of cynaroside, quercetin, ABTS, glucose, and sucrose, while exhibiting higher levels of total acid, malic acid, and citric acid. Three samples processed using SA-(MUSVFIR-RFV) formed another cluster, whereas samples dried using (CMC-Na)-(MUSVFIR-RFV)-40 % and (CMC-Na)-(MUSVFIR-RFV)-60 % were grouped into a separate cluster, both showing higher concentrations of soluble solids, TFC, and catechin. Notably, the USVFIR-RFV and (CMC-Na)-(MUSVFIR-RFV)-50 % samples were individually clustered into distinct groups, with a considerable distance between them, indicating significant compositional differences between these two drying methods.

Principal component analysis (PCA) enables dimensionality reduction of multi-dimensional data, effectively extracting key factors influencing cherry quality. It also provides a visual representation to intuitively illustrate the differences among various drying methods and the interrelationships between physicochemical properties. Fig. 6(b) presented the PCA results of the physicochemical quality of dried cherry products under different dehydration treatments. The results indicated that PC1 and PC2 accounted for 70.2 % and 12.1 % of the total variance, respectively, with a cumulative contribution rate of 82.3 %, which could better reflect the primary variations of each physicochemical properties of cherries. PC1 exhibited a significant positive correlation with cynaroside, quercetin, cyanidin-3-O-rutinoside, individual sugars, TPC, TFC, and antioxidant capacity. Whereas PC2 was mainly contributed by catechin, isochlorogenic acid, citric acid and organic acids. The positive and negative loading relationship between sugars and acids suggested that they play opposing roles in the regulation of flavour balance. Cherries with different drying process treatments were distributed farther in the PCA score plot and formed a clear grouping feature, which indicated that different dehydration treatments had a significant effect on the physicochemical quality of cherries. Analysis of Fig. 6(b) revealed that (CMC-Na)-(MUSVFIR-RFV) samples were distributed in the positive direction of PC1 and PC2 in different drying treatments, indicating higher physicochemical properties and flavour taste. Additionally, compared with RFV, USVFIR, and USVFIR-RFV drying methods, samples pretreated with CMC-Na and SA coatings followed by MUSVFIR-RFV segmented drying were all positioned in the positive direction of PC1. This suggested that coating treatment and segmented combined drying effectively reduce color degradation and the loss of flavor compounds, thereby enhancing the overall quality of dried cherries.

### 3.11 Correlation network analysis heatmaps

**Fig. 7:**
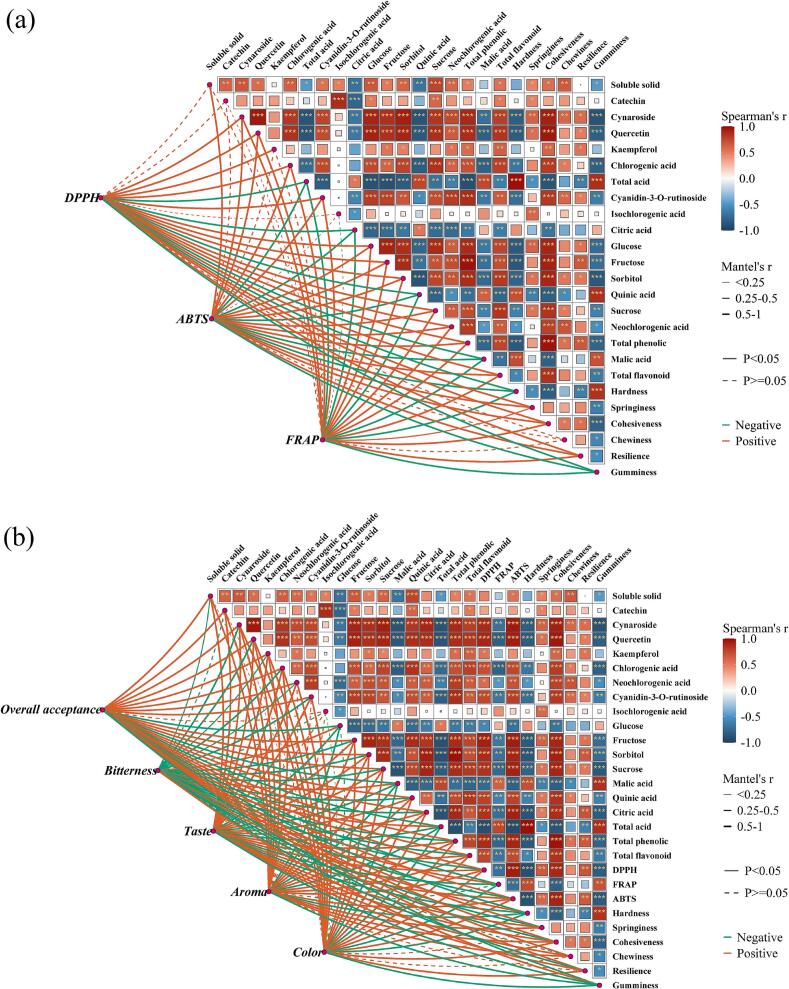
Correlation network analysis heatmaps between antioxidant capacity and sensory attributes, and various physicochemical properties of cherries under various drying processes.

Fig. 7(a) and (b) illustrate the correlation network analysis heatmaps, focusing on antioxidant capacity and sensory attributes, respectively, as the core research variables. The analysis integrated quality indicators such as soluble solids, sugars, organic acids, color parameters, and natural bioactive compounds in cherries, providing an in-depth exploration of the potential relationships and interactive mechanisms among quality factors during the drying process. In the heatmaps, blue represented negative correlations, while red indicated positive correlations. As shown in Fig. 7(a), antioxidant capacity exhibited a significant positive correlation (r > 0.8) with quercetin, cynaroside, chlorogenic acid, cyanidin-3-O-rutinoside, individual sugars, TPC, and TFC. This finding highlighted these compounds as key contributors to antioxidant capacity, emphasizing the importance of preserving these active substances during the drying process. Moreover, the significant correlations observed among TPC, TFC, and cyanidin-3-O-rutinoside further suggested the potential synergistic effects of these natural bioactive compounds during the drying process, collectively enhancing antioxidant capacity. A similar conclusion was reported by Shewale et al., [65] who found a direct relationship between TPC and antioxidant activity, providing additional support for the reliability of the present findings. Additionally, the negative correlations between malic acid, citric acid, and antioxidant capacity might be attributed to the oxidation or transformation reactions of acid compounds during drying. Analysis of Fig. 7(b) revealed a significant positive correlation (*P* < 0.05) between hardness, cohesiveness, and overall acceptability. This result could likely be attributed to the ability of textural properties to enhance the chewing experience, thereby improving consumers' overall perception. Additionally, TPC and cyanidin-3-O-rutinoside exhibited a strong positive correlation with color (r > 0.8), indicating that these natural pigments and antioxidant compounds play a critical role in maintaining the vibrant appearance of cherries during the drying process. Furthermore, taste and aroma were positively correlated with seven natural bioactive compounds (r > 0.75, *P* < 0.05), underscoring their contribution to sensory quality. Notably, glucose, organic acids, and gumminess were found to significantly contribute to bitterness, suggesting that the accumulation of these functional components may negatively affect sensory quality.

## 4 Conclusion

This study investigated the effects of CMC-Na and SA edible coating pretreatments and different moisture conversion points on the drying characteristics, physicochemical properties, texture, and sensory attributes of cherries dried using MUSVFIR-RFV segmented combination drying. The results demonstrated that, compared to traditional single-stage (RFV and USVFIR) drying methods, the MUSVFIR-RFV segmented drying technology exhibited significant advantages in improving drying efficiency, optimizing energy utilization, and maintaining fruit quality, with the application of edible coatings further enhancing these effects. Notably, the drying time of cherries subjected to the (CMC-Na)-(MUSVFIR-RFV) process was reduced by 30 ∼ 120 min compared to the USVFIR-RFV drying, further confirmed the ability of multi-frequency ultrasound to strengthen heat and mass transfer during drying. Physicochemical analyses revealed that CMC-Na and SA coating pretreatments effectively mitigated thermal-oxidative damage during the drying process, significantly enhancing the retention of anthocyanins, TPC, TFC, and natural antioxidant compounds in dried cherries. Cherries treated with the (CMC-Na)-(MUSVFIR-RFV)-50 % dehydration process exhibited color parameters closer to those of fresh samples. The antioxidant capacity results indicated that dried cherries processed with (CMC-Na)-(MUSVFIR-RFV) showed slightly higher DPPH, ABTS, and FRAP values compared to those treated with SA-(MUSVFIR-RFV). Additionally, the textural and sensory analyses revealed that the coated dried cherry products had better overall acceptability, along with a significant reduction in bitterness and off-flavors. In summary, the novel edible coating combined with multi-frequency ultrasonic segmented drying technology provides a scientific basis and technical support for energy-efficient and environmentally friendly drying of cherries by enhancing the energy efficiency of the drying process, optimizing heat and mass transfer, and protecting the physicochemical quality of the sample.

## CRediT authorship contribution statement

**Zepeng Zang:** Writing – review & editing, Writing – original draft, Supervision, Software, Resources, Methodology, Investigation, Formal analysis, Data curation, Conceptualization. **Xiaopeng Huang:** Writing – review & editing, Supervision, Resources, Methodology, Funding acquisition, Conceptualization. **Guojun Ma:** Writing – review & editing, Supervision, Resources, Project administration, Funding acquisition, Conceptualization. **Fangxin Wan:** Writing – review & editing, Validation, Supervision, Methodology, Formal analysis, Conceptualization. **Yanrui Xu:** Validation, Software, Methodology, Conceptualization. **Qiaozhu Zhao:** Validation, Methodology, Investigation, Data curation, Conceptualization. **Bowen Wu:** Validation, Methodology, Data curation. **Hongyang Lu:** Software, Methodology, Data curation. **Zelin Liu:** Validation, Formal analysis, Data curation.

## Declaration of competing interest

The authors declare that they have no known competing financial interests or personal relationships that could have appeared to influence the work reported in this paper.
